# Chronic beta-adrenergic blockade prevents volume overload-induced re-localization and oxidation of soluble guanylyl cyclase

**DOI:** 10.1186/2050-6511-14-S1-O22

**Published:** 2013-08-29

**Authors:** Yuchuan Liu, Louis Dell’Italia, Victor Rizzo, Emily J Tsai

**Affiliations:** 1 Cardiovascular Research Center, Temple University School of Medicine, Philadelphia, PA, 19140, USA; 2 Division of Cardiovascular Disease, Department of Medicine, University of Alabama Birmingham School of Medicine, Birmingham, AL, 35233, USA; 3 Department of Anatomy and Cell Biology, Temple University School of Medicine, Philadelphia, PA, 19140, USA; 4 Section of Cardiology, Department of Medicine, Temple University School of Medicine, Philadelphia, PA, 19140, USA

## Background

While β-adrenergic blockade is a cornerstone of heart failure therapy, its therapeutic role in chronic mitral regurgitation remains questionable. Animal studies and a small clinical trial have demonstrated cardiac functional improvement with β_1_-adrenoceptor blocker metoprolol in chronic mitral regurgitation [1,2]. How β_1_AR-blockade halts functional decline of the volume-overloaded, eccentric hypertrophied heart is not well understood; anti-oxidant effects of β-blockade (βB) may play a role. We recently demonstrated that volume-overload cardiac stress induces re-localization and microdomain-specific oxidation of the nitric oxide receptor soluble guanylyl cyclase (sGC) in the failing heart [3,4]. Given that nitric oxide-cyclic guanosine monophosphate (NO-cGMP) modulates cardiac contractility and protects against cardiac hypertrophy, we hypothesized that β_1_AR-blockade prevents oxidation of sGC and promotes myocardial NO-cGMP signaling in a microdomain-specific fashion.

## Materials and methods

Volume-overload (VO) was established by chordal rupture-induced mitral regurgitation (MR) in mongrel dogs. Some dogs were treated with metoprolol succinate (100mg orally once daily; MR+ βB). Expression, localization, cyclase activity, and redox state of myocardial sGC were assessed in Control, MR, and MR+βB dogs.

## Results

sGCα_1_ and -β_1_ subunits were detected within and outside of caveolae-enriched lipid rafts (Cav3^+^LR). In MR, total sGCα_1_ expression fell to nearly 50% of Control and re-localized away from Cav3^+^LR to non-lipid raft microdomains (NLR). While overall sGCβ_1_ expression was also less in MR+βB, caveolae-localization of sGCβ_1_ was preserved. Overall NO-responsiveness of sGC remained intact in MR hearts, irrespective of βB therapy. However, a potentiated response to heme/NO-independent sGC activator BAY 60-2770 suggested that a subset of sGC was heme-oxidized in MR but not in Control or MR+βB. Moreover, differential responses to BAY 60-2770 and NO were noted in Cav3^+^LR and NLR microdomains. In Control hearts, responses to BAY 60-2770 and NO were similar within respective microdomains, suggesting a predominantly reduced form of sGC in both Cav3^+^LR and NLR of Controls. In contrast, BAY 60-2770 response of NLR-localized sGC was potentiated in MR but not in MR+βB hearts, suggesting that βB therapy prevented oxidation of NLR-localized sGC. Moreover, BAY 60-2770 responses of Cav3^+^LR-localized sGC were not potentiated in any hearts, suggesting an anti-oxidation protection associated with caveolae-localization. These changes in caveolae-localization and redox state of sGC were also reflected by microdomain distribution of VASP phosphorylation.

## Conclusion

β_1_AR blocker mediated cardioprotection in the volume-overloaded heart is associated with enhanced microdomain specific myocardial NO-cGMP signaling, both within and outside of caveolae. Such prevention of volume overload-induced spatial and redox dysregulation of myocardial sGC suggests novel strategies to enhancing cardioprotective NO-cGMP signaling.
